# A Case of Covid-19 Respiratory Illness with Subsequent Seizure and Hemiparesis

**DOI:** 10.31661/gmj.v9i0.1915

**Published:** 2020-07-12

**Authors:** Ava Hamidi, Behnam Sabayan, Frazaneh Sorond, Alexander J Nemeth, Afshin Borhani-Haghighi

**Affiliations:** ^1^Clinical Neurology Research Center, Shiraz University of Medical Sciences, Shiraz, Iran; ^2^Department of Neurology, Northwestern University Feinberg School of Medicine, Chicago, IL, USA; ^3^Department of Radiology, Northwestern University Feinberg School of Medicine, Chicago, IL, USA

**Keywords:** Covid-19, Severe Acute Respiratory Syndrome Coronavirus 2 (S, Brain, Stroke, Cerebrovascular Accident

## Abstract

**Background:**

Neurological manifestations and complications are common in viral infections, and they are significant sources for clinical deterioration and poor clinical outcomes. **Case report:** The current report presents a 38year-old man with Covid-19 respiratory illness who subsequently developed neurological complications and clinical worsening leading to death. This patient sought medical attention after five days of progressive cough, fever, and dyspnea. On arrival in the emergency room, he was found to have hypoxic respiratory failure resulting in intubation and intensive care unit (ICU) admission. Chest CT scan was characteristic for Covid-19 infection, and PCR test on tracheal samples confirmed the diagnosis. On day nine of admission, he developed generalized tonic colonic seizure associated with deterioration of mental status and hemiparesis. Repeated brain CT scans showed subcortical hypoattenuation with associated sulcal effacement in the left occipital and posterior parietal lobes concerning for ischemic changes. The patient passed away on day 17 despite supportive measures.

**Conclusion:**

This observation and recent evidence on Covid-19 CNS involvement highlight the need for further studies on early recognition of neurological complications in Covid-19 patients.

## Introduction

Coronavirus disease 2019 (COVID-19) has affected more than 1.5 million people worldwide with significant disruptions in the health care systems [1]. The novelty of the virus, rapid pace of spread, and considerable case fatality and morbidity require better characterization of the clinical features and determination of multi-system complications [2]. Neurological manifestations and complications are common in viral infections, and they are a major source for clinical deterioration and poor clinical outcomes. This report presents a case of Covid-19 respiratory illness with subsequent seizures and brain ischemic changes.

## Case Presentation

Our patient is a 38-year-old Caucasian man living in southern Iran with a history of childhood asthma, who presented with five days of progressive cough, fever, and dyspnea. He stopped smoking seven years ago, and he is a past opium user currently on maintenance methadone therapy. On arrival in the emergency room (ER), the temperature was 39 °C, heart rate 100 beats/min, blood pressure 110/70 mmHg, and he was in severe respiratory distress. Due to hypoxic respiratory failure, he was sedated and intubated in the ER. CBC showed leukocytosis (10400 per mm3) with mild lymphopenia at 11.3 %. The patient had elevated liver enzymes (AST: 870, ALT: 660 units/liter) and serum C reactive protein (3+). The hematological evaluation was significant for INR 1.8. Kidney function and electrolytes were within normal limits. The patient was admitted to the intensive care unit (ICU) with high clinical suspicion for Covid-19 infection. - Tracheal samples were transported to the laboratory. Then, he received oseltamivir 75 mg and hydroxychloroquine 200mg twice a day. His maintenance methadone dose was continued. Supplementary Figure-1 (S-1) shows initial chest CT with findings consistent with Covid-19 infection [3]. The patient remained febrile and ventilator dependent. On day 5, Amikacin and Meropenem were added for persistent fevers and lack of clinical improvement. Despite initial improvements, on day 9, he developed a brief generalized-tonic-colonic seizure with post-ictal reduction in left-sided more than right-sided movements. He was given diazepam and loaded with Levetiracetam. Antibiotics were modified due to concerns for lowering seizure threshold. On neurological examination, his cranial nerves were intact. He had left-sided hemiplegia and decreased spontaneous movements on the right-side but was able to localize to noxious stimulation. Initial Brain CT after seizure did not show significant abnormalities (Figure-1-A). Transthoracic echocardiography showed an ejection fraction of 55% with no evidence of endocarditis, myocarditis, or pericarditis. Two separate sets of blood cultures were negative. The next day, he had another generalized-tonic-colonic seizure lasting for a minute with further deterioration in his mental status and weakness. The anti-epileptic regimen was modified, and he underwent a follow up brain CT scan. We found subcortical hypoattenuation with associated sulcal effacement in the left occipital and posterior parietal lobes concerning for ischemic changes (Figure-1-B). Further brain and vascular imaging could not be completed due to unstable medical conditions. The patient remained unresponsive despite weaning sedation. Follow up CT chest presented in Supplementary Figure-2 (S-2). Broad-spectrum antimicrobial therapy was continued. Due to persistently elevated INR, a lumbar puncture was not performed. Hepatitis panel and HIV testing were negative. Continuous EEG monitoring was not available at the medical facility, and the patient was unstable for transfer to a higher-level care center. Covid-19 PCR was reported positive. On day 17 of admission, a cardio-pulmonary code was called, and the patient passed away. No autopsy was performed.

## Discussion

Covid-19 can potentially involve the brain in various ways such as meningoencephalitis, cytokine release syndrome, and venous or arterial vascular events secondary to a hypercoagulable state and cardiac abnormalities [4]. Poyiadji and colleagues recently reported a Covid-19 patient with altered mental status who had acute hemorrhagic necrotizing encephalopathy. It was raising the possibility of a blood-brain-barrier breakdown in response to a severe systemic inflammatory state [5].Similarly, Moriguchi and colleagues presented the first case of meningitis associated with Covid-19, who developed convulsion and encephalopathy as initial manifestations [6].Besides, a growing evidence indicates that Covid-19 patients have profound coagulation abnormalities putting them at risk for ischemic events. Zhang and colleagues recently reported three cases with multi-focal brain infarcts who had clinically significant coagulopathy and antiphospholipid antibodies [7].Such clinical observations highlight the need for further studies on early recognition of neurological complications in Covid-19 patients. Our case received care under a limited resource situation during the peak of the Covid-19 outbreak in Iran, which has put a significant strain on the country’s health care system. Such data may reflect the reality of care in many parts of the world with the current pandemic and underscores the importance of utilizing easily accessible clinical tools for risk stratification of Covid-19 patients. Over a short time, the world has witnessed an increasing number of Covid-19 patients requiring ICU admissions, where intubation and sedation can potentially delay recognition of acute neurological complications. Neurological complications such as seizure, encephalitis, and cerebrovascular events need to be considered in Covid-19 patients who experience deterioration in their clinical course.

## Conclusion

 This observation and recent evidence on Covid-19 CNS involvement highlight the need for further studies on early recognition of neurological complications in Covid-19 patients.

## Acknowledgement

 We would like to thank Dr. Houshmandi and Ms. Zafarmnad for their assistance.

## Conflict of Interest

 The authors have no conflict of interest.

**Figure 1A:**
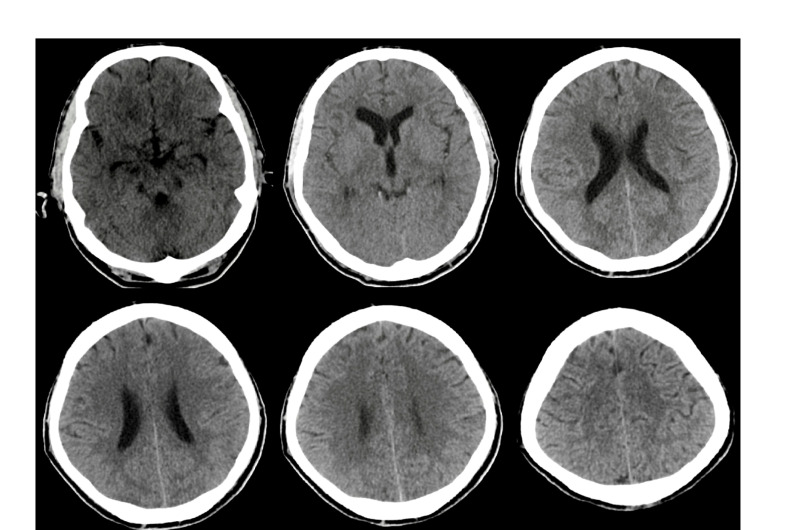
First brain CT scan after developing first seizure.

**Figure 1B:**
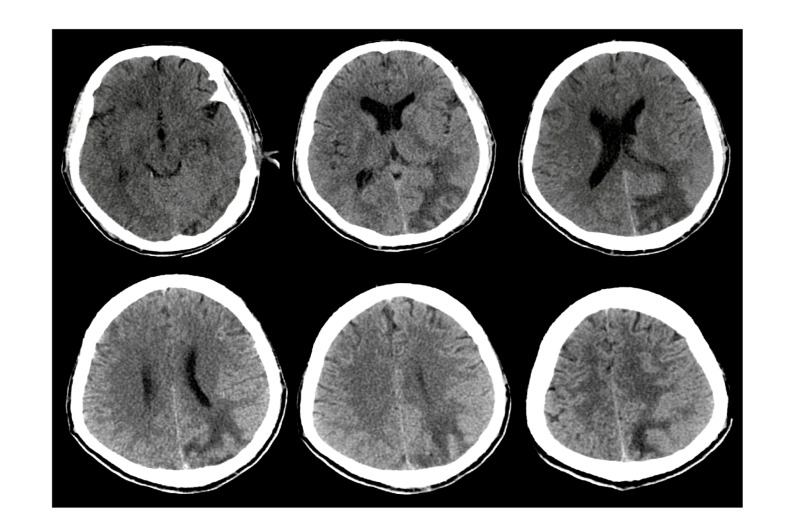
Development of subcortical hypoattenuation with associated mass effect including sulcal effacement in the left occipital and left parietal lobes. No hyperdense venous sinuas is identified.
